# The Neuroprotective Effect of Short Chain Fatty Acids Against Sepsis-Associated Encephalopathy in Mice

**DOI:** 10.3389/fimmu.2021.626894

**Published:** 2021-01-28

**Authors:** Jiaming Liu, Yangjie Jin, Yanglie Ye, Yahui Tang, Shanshan Dai, Mengfang Li, Guangju Zhao, Guangliang Hong, Zhong-Qiu Lu

**Affiliations:** ^1^ Department of Preventive Medicine, School of Public Health and Management, Wenzhou Medical University, Wenzhou, China; ^2^ Department of Emergency Medicine, The First Affiliated Hospital of Wenzhou Medical University, Wenzhou, China

**Keywords:** sepsis-associated encephalopathy, short chain fatty acids, neuroinflammation, behavioral impairment, neuroprotection

## Abstract

Short chain fatty acids (SCFAs) are known to be actively involved in multiple brain disorders, but their roles in sepsis-associated encephalopathy (SAE) remain unclear. Here, we investigated the neuroprotective effects of SCFAs on SAE in mice. Male C57BL/6 mice were intragastrically pretreated with SCFAs for seven successive days, and then subjected to SAE induced by cecal ligation and puncture. The behavioral impairment, neuronal degeneration, and levels of inflammatory cytokines were assessed. The expressions of tight junction (TJ) proteins, including occludin and zoula occludens-1 (ZO-1), cyclooxygenase-2 (COX-2), cluster of differentiation 11b (CD11b), and phosphorylation of JNK and NF-κB p65 in the brain, were measured by western blot and Immunofluorescence analysis. Our results showed that SCFAs significantly attenuated behavioral impairment and neuronal degeneration, and decreased the levels of IL-1β and IL-6 in the brain of SAE mice. Additionally, SCFAs upregulated the expressions of occludin and ZO-1 and downregulated the expressions of COX-2, CD11b, and phosphorylation of JNK and NF-κB p65 in the brain of SAE mice. These findings suggested that SCFAs could exert neuroprotective effects against SAE in mice.

## Introduction

Septic-associated encephalopathy (SAE) is one of the main sequelae of sepsis survivors, affecting between 8-70% of patients (1, 2). SAE increases the risk of mortality of patients with sepsis (3). The pathophysiology of SAE is complex; blood–brain barrier (BBB) impairment and neuroinflammation might be the key processes in the occurrence of SAE (4–6). BBB could control the balance of blood–brain water, molecules, and ion, and restrain the invasion of immune cells, toxins, and pathogens. Of note, sepsis is often accompanied by increased permeability of BBB, which leads to SAE. Following BBB impairment, neuronal degeneration and brain edema aggravate brain injuries and neuroinflammation (7, 8). Dysfunction of the vascular complex, including endothelial cells, astrocytes, and the blood-brain barrier, and activation of microglia result in neuroinflammation. Neuroinflammation in sepsis develops with the activation of brain endothelial cells, the increase of BBB permeability, and the increase of neutrophil infiltration; these abnormalities can lead to brain dysfunction (9). Microglia could be activated in animal models (10, 11) and patients with sepsis (12, 13). So far, aside from the application of antimicrobial therapy and timely source control in sepsis, there are no effective intervention measures to prevent post-sepsis neurological dysfunctions. Therefore, it is urgent to explore neuroprotective agents to attenuate SAE.

Recent studies have emphasized the critical impact of natural products on brain disorders. There is increasing evidence that short chain fatty acids (SCFAs) are known to be actively involved in multiple brain disorders. SCFAs including acetate, propionate, and butyrate are produced by gut microflora metabolizing dietary fiber. SCFAs could travel from the gut to the brain to perform a number of effects (14) and modulate CNS functions, including brain development and behavior (15, 16). Remarkably, our studies have shown that the levels of SCFAs in SAE model mice were significantly lower than those of the control group (**Supplementary Figure 1**). Our previous study demonstrated that administration of exogenous SCFA acetate could attenuate cognitive impairment and decrease the expression of microglial markers (CD11b) in an Alzheimer’s disease model mice. Moreover, our previous study demonstrated that SCFA butyrate could prevent dopaminergic degeneration and attenuate the disruption of BBB in Parkinson’s disease model animals. Additionally, SCFA butyrate could reverse the traumatic-brain-injury-induced decrease of tight junction (TJ)-associated proteins, such as occludin and ZO-1. These considerations suggest that SCFAs might help prevent the behavioral impairment and neuroinflammation found in SAE.

In this study, we explored the effects and underlying mechanisms of SCFAs on SAE. We revealed that SCFAs’ pretreatment improved the behavioral dysfunction of SAE model mice. SCFAs also significantly attenuated BBB impairment and neuroinflammation of sepsis mice. In addition, we further confirmed that SCFAs could decrease excessive activation of microglia and production of pro-inflammatory cytokines, and suppress phosphorylation levels of JNK and NF-κB p65 in the brain of SAE mice. Thus, SCFAs might be a novel dietary supplementation for the prevention of SAE.

## Materials and Methods

### Animal

Male C57BL/6 mice (22 ± 2 g, 6 - 8 weeks old) were purchased from the SLAC Laboratory Animal Co., Ltd, Shanghai. All animals were raised in pathogen-free cages at the Experimental Animal Center of Wenzhou Medical University in a controlled temperature (22 ± 1°C) environment with relative humidity (65 ± 5%). The mice were housed under a light/dark cycle, and water and food were available ad libitum during the experiment. All experiments were carried out in accordance with the Guide for Animal Experimentation of Wenzhou Medical University and approved by the Animal Experimentation Ethics Committee of Wenzhou Medical University.

### Induction of SAE Mouse Model

SAE was induced in mice by cecal ligation and puncture (CLP) procedure according to a previous study with a minor modification (17). Mice were anesthetized intraperitoneally with 350 mg/kg chloral hydrate and placed on the operating table. The abdomen was scraped clean and sterilized with alcohol. The midline skin incision was made about 1.5 - 2 cm long, and then the cecum was separated. About 50% of the cecum was ligated from the surface of the anti-mesentery with a 21-gauge needle and the mesentery vessel was protected. A small amount of feces was squeezed out of the intestine and the cecum was moved back to the abdominal cavity. The incision was closed with surgical suture 4 - 0 and then the mice were resuscitated by subcutaneous injection of normal saline (5 mL/100g body weight). In the sham operation group, only laparotomy was performed without cecal ligation or perforation.

### Drug Pretreatment and Experimental Design

The animals were randomly divided into three groups: Sham group, SAE group, and SAE + SCFAs group. The Sham group received the sham operation without drug pretreatment; the SAE group received the CLP surgery and an equal volume of saline. The SAE + SCFAs group received the CLP surgery and SCFAs pretreatment. SCFAs (acetate, propionate, and butyrate) were purchased from Aladdin, Co. Ltd., China. SCFAs (acetate: propionate: butyrate at a ratio of 3: 1: 1) at 500 mg/kg body weight were administrated intragastrically twice a day for seven consecutive days before CLP surgery.

### SHIRPA Test

The SmithKline/Harwell/Imperial College/Royal Hospital/Phenotype Assessment (SHIRPA) was an effective method to study the dysfunction of the central nervous system (CNS) in mice (18). The SHIRPA protocol was used to assess mouse behavioral changes in mice during sepsis. SHIRPA consisted of a series of 40 simple tests and was divided into five functional categories, as described by Jeremias et al. (19). At 12 h after the CLP operation, the SHIRPA test was started with the viewing jar (diameter 11 cm, height 25 cm). The mice were placed in the viewing jar for 5 min to observe defecation, urination, respiration rate, body position, and spontaneous activity. Then the mice were transferred to the arena, a 55 × 33 × 18 cm box with a 11 × 11 cm square grid at the bottom. A series of tests on motor behavior, autonomous function, and muscle tone and strength were carried out. Five domain scores and one total score were recorded.

### Fluoro-Jade C Staining

Fluoro-Jade C (FJC) staining was found to stain all degenerated neurons, whether through specific injury or cell death mechanism. The Fluoro-Jade C staining procedure was performed as in a previous study (20). At 12 h after the CLP operation, the mice were euthanized and the brain samples were fixed with 4% polyformaldehyde for two days. Then the fixed samples were dehydrated with ethanol, soaked in paraffin, and made into paraffin blocks. After slicing, the brain sections were rehydrated in a series of reduced gradient ethanol, and then immersed in a 0.06% potassium permanganate solution for 10 min. Subsequently, the slices were transferred to a 0.0001% FJC (Millipore, Darmstadt, Germany) working fluid for 20 min. The sections were sealed and observed with fluorescence microscope (Leica Microsystems, Wetzlar, Germany).

### Immunofluorescence

The mice were sacrificed at 12 h after the CLP operation, and the brain samples were taken, embedded in paraffin wax, and then cut into 5 µm sections by rotary microtome. After dewaxing and rehydrating, the sections were blocked with 5% fetal bovine serum (FBS) and incubated with primary antibodies (Occludin, ZO-1 and CD11b) overnight at 4°C. The antibodies details were as fellow: Occludin (1: 200, Proteinch, Rosemont, IL, USA), ZO-1 (1: 200, Santa Cruz Biotechnology, Dallas, Texas, USA), and CD11b (1: 200, Bioworld Technology, Bloomington, MN, USA). The sections were washed three times in PST and incubated with Alexa fluor 488 anti-mouse secondary antibody (Invitrogen Life Technologies, Carlsbad, CA, USA) or Alexa fluor plus 546 anti-rabbit secondary antibody (Invitrogen Life Technologies, Carlsbad, CA, USA) at 37°C for 30 min. Cell nuclei were stained with DAPI (SouthernBiotech, Birmingham, AL, USA) at room temperature (RT) for 10 min. The sections were sealed with glycerin and observed under fluorescence microscope (Leica Microsystems, Wetzlar, Germany).

### Western Blot

The mice were deeply anesthetized and sacrificed at 12 h after the CLP operation. The brain samples were quickly harvested and stored at -80°C for further use. The samples were cracked with RIPA lysis buffer (Beyotime Biotechnology, Shanghai, China) and the homogenate was centrifuged at 12, 000 × g at 4 °C for 20 min. The centrifugal supernatant was treated with BCA kit (Beyotime Biotechnology, Shanghai, China) and the absorbance were measured at 540 nm to draw the standard curve. The protein concentration of the samples was controlled at 2 µg/µL. Equal protein (10 µg) was added to the 10% SDS-PAGE and electrotransferred to a nitrocellulose (NC) membrane (Millipore, MA, USA). The membrane was immersed in 5% skimmed milk at RT for 2 h and then incubated in primary antibodies (Occludin, ZO-1, COX-2, CD11b, JNK, JNK1/2/3 (phosphor-T183/Y185), NF-κB p65, phosphor-NF-κB p65) overnight at 4°C. The antibodies’ details were as follows: Occludin (1: 1000, Proteinch, Rosemont, IL, USA), ZO-1 (1: 1000, Santa Cruz Biotechnology, Dallas, Texas, USA), COX-2 (1: 1000, Bioworld Technology, Bloomington, MN, USA), CD11b (1: 1000, Bioworld Technology, Bloomington, MN, USA), JNK (1: 1000, Bioworld Technology, Bloomington, MN, USA), JNK1/2/3 (1: 1000, Bioworld Technology, Bloomington, MN, USA), NF-κB p65 (1: 1000, Bioworld Technology, Bloomington, MN, USA), phosphor-NF-κB p65 (1: 1000, Bioworld Technology, Bloomington, MN, USA), and β-actin (1: 5000, Bioworld Technology, Bloomington, MN, USA). The membrane was washed in PBST and incubated in HRP conjugated secondary antibody (1: 5000, Beyotime Biotechnology, USA) at RT for 1 h. The membrane was imaged in the Western Bright-ECL gel recording system (Bio-Rad, USA). β-actin was used as a loading control.

### ELISA Assay

The brain samples were collected immediately. Protein was extracted by homogenizing in ice-cold RIPA lysis buffer with a mix of the protease inhibitor PMSF. The homogenate was centrifuged at 12, 000 × g for 20 min, and then the supernatant was obtained. The levels of IL-1β and IL-6 in brain tissue were measured by ELISA kit (Multi Sciences, China). The standard curve was constructed to calculate the concentrations of IL-1β and IL-6 in test samples. Values were expressed as pg/mg.

### SCFAs Analysis

The colon contents (100 mg) were added with 1ml water (0.5% phosphoric acid and 50 μg/ml 2-ethylbutyric acid). The following steps were performed: freezing and grinding, ice water bath ultrasound for 30min, standing at 4 °C for 30min, 13,000×g centrifugation for 15min (4 °C), adding 500 μl ethyl acetate to the supernatant, vortex mixing, ice water bath ultrasound for 10min, 13, 000 × g centrifugation for 10min. The supernatant was analyzed (Agilent Technologies Inc. CA, UAS). The chromatographic conditions were as follows: HP FFAP capillary column (Agilent J&W Scientific, Folsom, CA, USA), helium as carrier gas, flow rate of 1.0 ml/min, injection temperature of 260 °C. The injection volume was 1 μL. Mass spectrometry conditions were as follows: electron bombardment ion source, ion source temperature 230 °C, quadrupole temperature 150 °C, transmission line temperature 230 °C, electron energy 70 eV.

### Statistical Analysis

Statistical analysis was carried out in SPSS statistics V19.0 software. All data were analyzed by one-way ANOVA and tested by Newman Keuls. Values were presented as mean ± stand error of mean (SEM). *P* < 0.05 was considered to be significant.

## Results

### Effect of SCFAs on the Survival Rate in SAE Mice

The survival rate was shown in **Supplementary Figure 2**. The 7-day survival rate in the Sham group was almost 100%. At seven days after the CLP operation, the survival rate (50%) was lower than that of the Sham group. SCFAs decreased the survival rate compared with SAE, but did not reach a statistical significance.

### SCFAs Ameliorated Behavioral Impairment in SAE Mice

**Figure 1:**
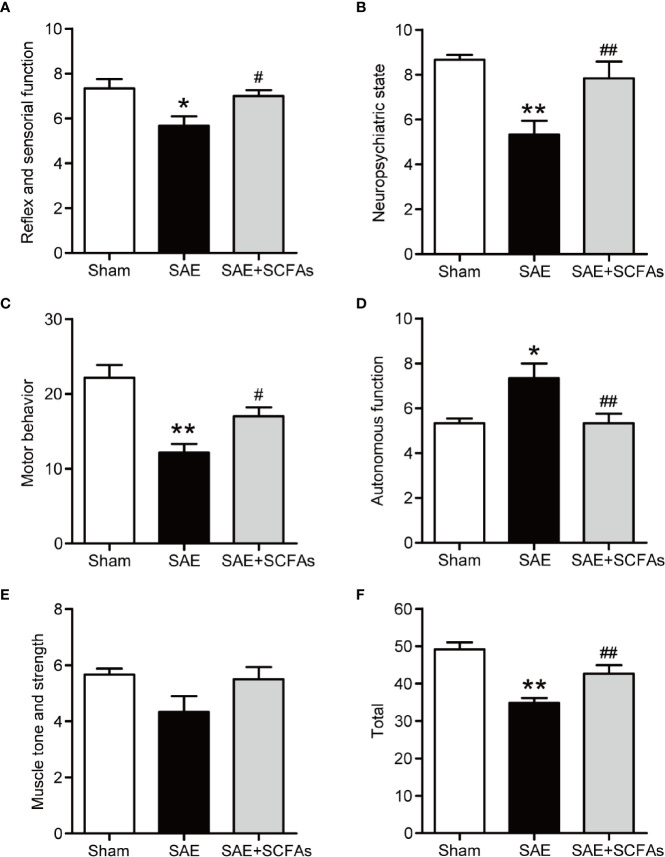
Effect of SCFAs on behavioral impairment in SAE mice. **(A–F)** Effect of SCFAs on the behavioral impairment at 12 h after CLP operation. Behavioral impairment was measured by the SHIRPA protocol in the five distinct functional categories. The sum of the scores of five functional categories was shown as the “total” score. Error bars indicate mean ± SEM. ^*^P < 0.05 versus Sham group, ^**^P < 0.01 versus Sham group, *^#^P* < 0.05 versus SAE group, *^##^P* < 0.01 versus SAE group.

Behavior changes in five different functional categories were shown in **Figures 1A–F** at 12 h after the CLP operation. The total score of the five functional categories was displayed as total scores. Compared with Sham mice, the SAE mice showed a decrease in reflex and sensory function, neuropsychiatric state, and motor behavior, which was reversed by SCFAs (**Figures 1A–C**). The autonomic function of SAE mice was significantly higher than that of Sham mice, while that of SAE + SCFAs mice was significantly lower (**Figure 1D**). There was no significant difference in muscle tone and strength among the three groups (*P* > 0.05, **Figure 1E**). The total score of SAE mice was significantly lower than that of Sham mice (*P* < 0.01, **Figure 1F**). However, the total score of SAE + SCFAs mice was significantly higher than that of SAE mice (*P* < 0.01, **Figure 1F**), suggesting that SCFAs could reduce behavioral disorder in SAE.

### SCFAs Ameliorated Neuronal Degeneration in SAE Mice

**Figure 2:**
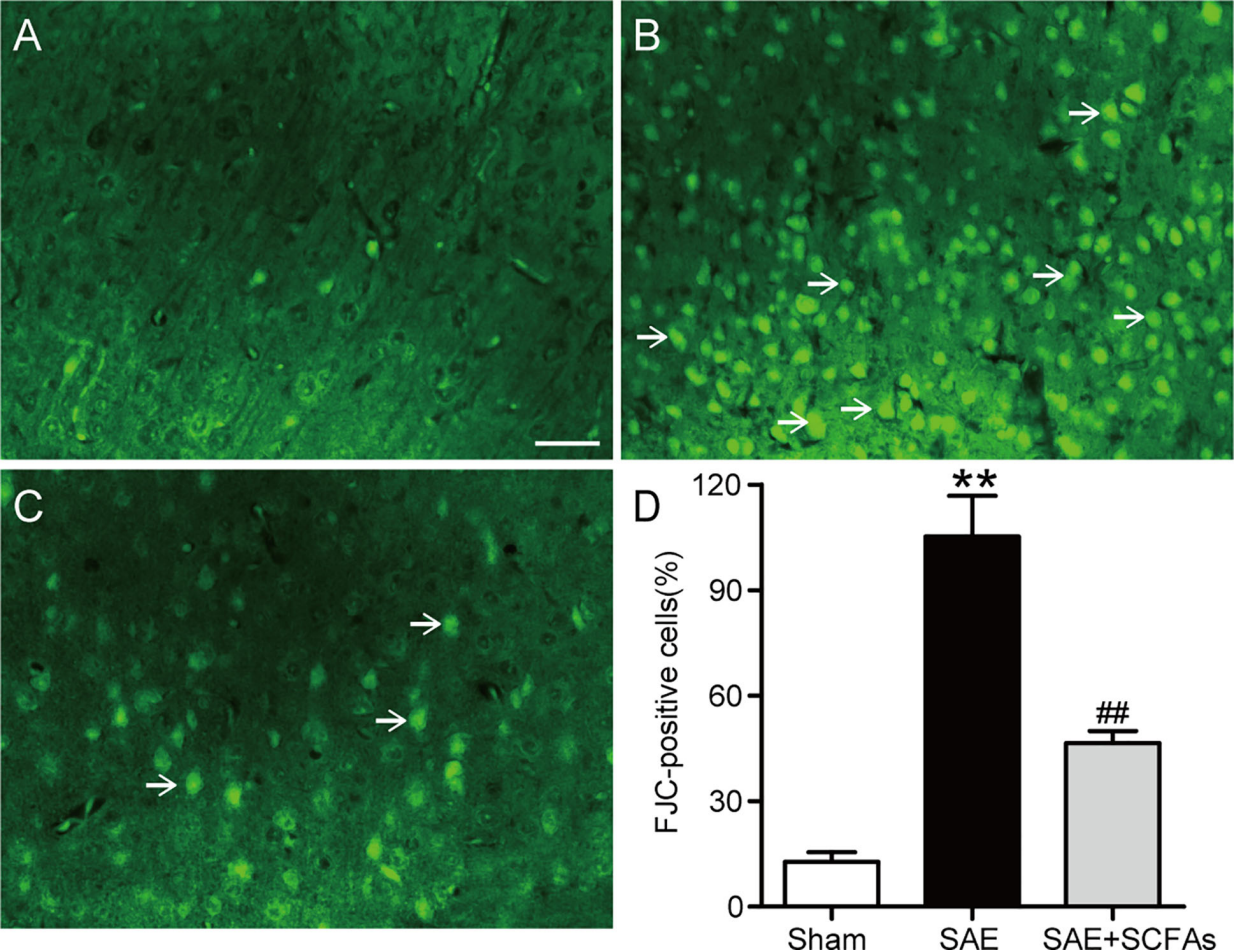
Effect of SCFAs on neuronal degeneration in SAE mice. **(A–C)** Representative images of immunofluorescence for degenerating neurons (green). **(A)** Representative images of immunofluorescence in the Sham group, **(B)** Representative images of immunofluorescence in the SAE group, **(C)** Representative images of immunofluorescence in the SAE+SCFAs group. The degenerating neurons were determined by FJC staining (green). The arrows indicated FJC-positive cells. Magnification 200 ×. Scale bar = 100 µm. **(D)** Quantification of immunofluorescence for FJC-positive cells. Error bars indicate mean ± SEM. ^**^ *P* < 0.01 versus Sham group, *^##^P* < 0.01 versus SAE group.

At 12 h after the CLP operation, the degeneration of neurons was observed by FJC staining. The number of FJC-positive neurons was counted and used as an indicator of the severity of neuron degeneration. In **Figure 2**, a large number of FJC-positive neurons were detected in SAE mice, while there was almost no degeneration in Sham mice (*P* < 0.01). However, the number of FJC-positive neurons in SAE + SCFAs mice was significantly less than that in SAE mice (*P* < 0.01).

### SCFAs Ameliorated BBB Disruption in SAE Mice

**Figure 3:**
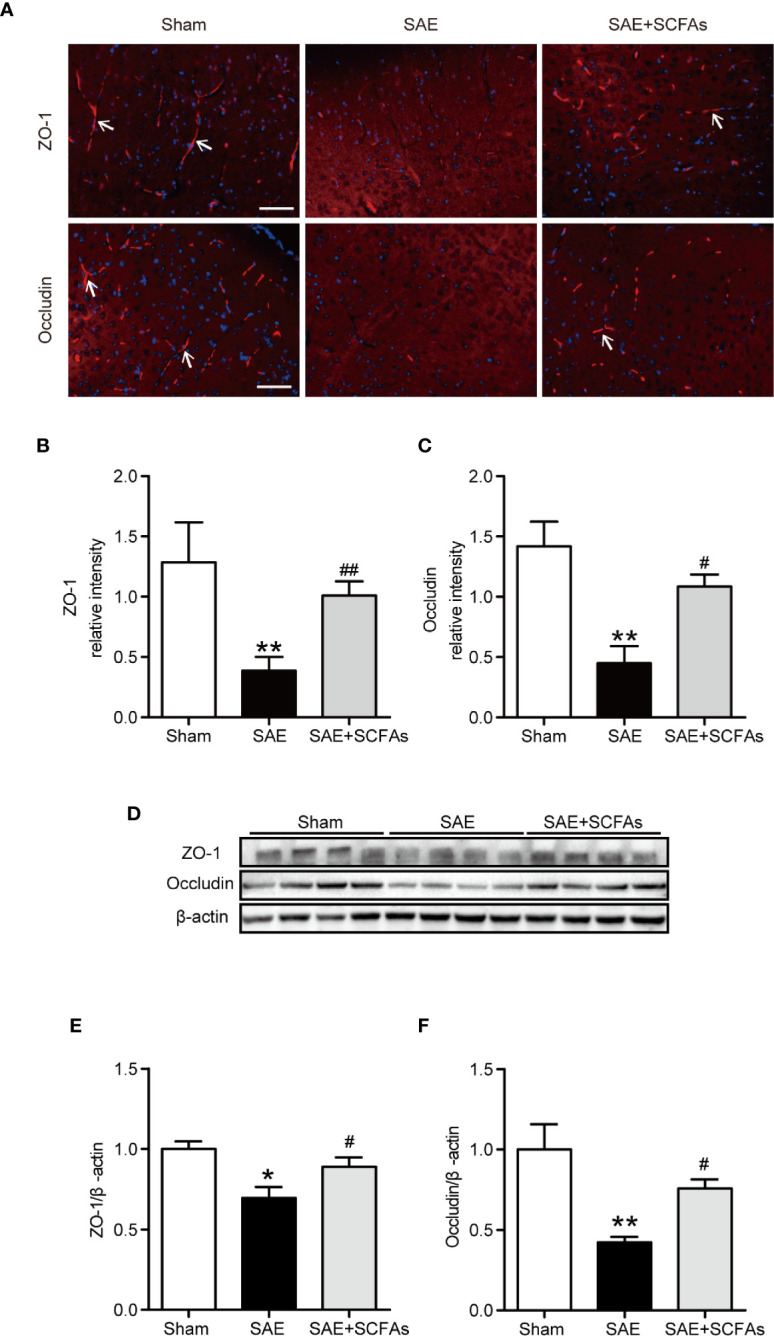
Effect of SCFAs on the levels of Occludin and ZO-1 in SAE mice. **(A)** Representative images of immunofluorescence for Occludin and ZO-1. The arrows indicated positive proteins. Magnification 400 ×. Scale bar = 50 µm. **(B**, **C)** Quantification of immunofluorescence data for Occludin and ZO-1 proteins. **(D)** Western blot analysis of Occludin and ZO-1 expression. **(E**, **F)** Quantitative analysis of expression of Occludin and ZO-1; the reference value was normalized to the Sham group. n = 4 per group. Error bars indicate mean ± SEM. ^*^P < 0.05 versus Sham group, ^**^P < 0.01 versus Sham group, *^#^P* < 0.05 versus SAE group, *^##^P* < 0.01 versus SAE group.

To evaluate the integrity of BBB, the levels of ZO-1 and Occludin were measured. The fluorescence signal intensity of ZO-1 and Occludin in the SAE group were lower than those in Sham group (ZO-1: *P* < 0.01, **Figures 3A, B**; Occludin: *P* < 0.01, **Figures 3A, C**). However, the SAE + SCFAs group showed higher intensity than those in the SAE group (ZO-1: *P* < 0.01, **Figures 3A, B**; Occludin: *P* < 0.05, **Figures 3A, C**). Detected by western blot, the levels of ZO-1 and Occludin were significantly lower than those in the Sham group, while the levels of ZO-1 and Occludin were significantly increased in the SCFAs + SAE group than those in the SAE group (**Figures 3D–F**).

### SCFAs Ameliorated Neuronal Inflammation in SAE Mice

**Figure 4:**
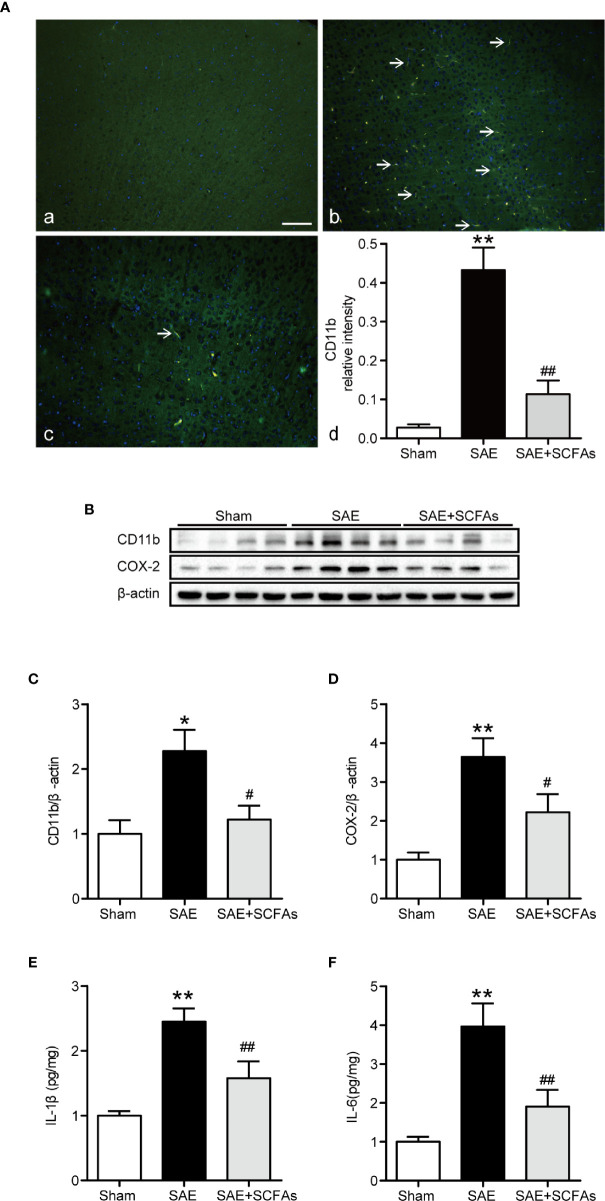
Effect of SCFAs on the neuronal inflammation in SAE mice. **(A)** Representative images of immunofluorescence for CD11b. **(A)** Representative images of immunofluorescence in the Sham group. **(B)** Representative images of immunofluorescence in the SAE group. **(C)** Representative images of immunofluorescence in the SAE + SCFAs group. The arrows indicated positive proteins. Magnification 400 ×. Scale bar = 50 µm. **(D)** Quantification of immunofluorescence data for CD11b proteins. n = 4 per group **(B)** Western blot analysis of expressions of CD11b and COX-2. **(C**, **D)** Quantitative analysis of CD11b and COX-2 expression, the reference value was normalized to the Sham group, n = 4 per group. **(E**, **F)** The levels of IL-1β and IL-6 in the brain, the reference value was normalized to the Sham group, n = 4 - 8 per group. Error bars indicate mean ± SEM. ^**^P < 0.01 versus Sham group, *^#^P* < 0.05 versus SAE group, *^##^P* < 0.01 versus SAE group.

**Figure 5:**
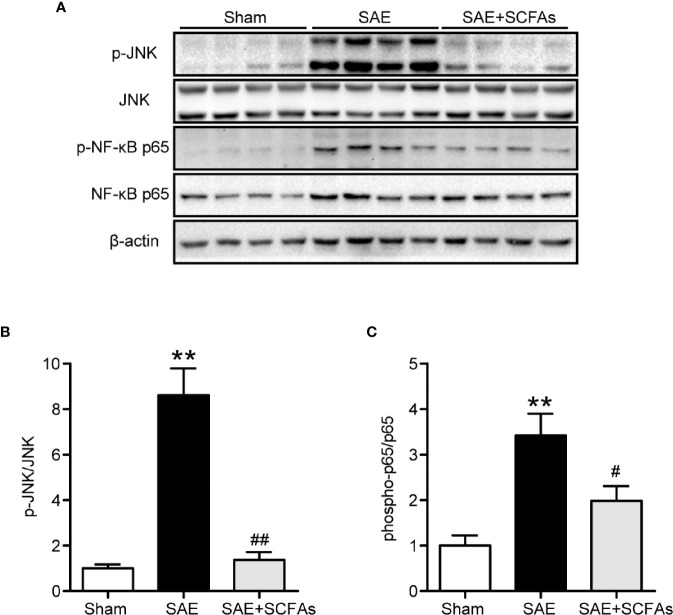
Effect of SCFAs on JNK and NF-κB p65 signaling activity in SAE mice. **(A)** Western blot analysis of expressions of p-JNK and JNK, and phosphor-NF-κB p65 and NF-κB p65. **(B**, **C)** Quantitative analysis of p-JNK/JNK and phosphor-NF-κB p65/NF-κB p65 expression, the reference value was normalized to the Sham group. n = 4 per group. Error bars indicate mean ± SEM. ^**^P < 0.01 versus Sham group, *^#^P* < 0.05 versus SAE group, *^##^P* < 0.01 versus SAE group.

Neuronal inflammation plays an important role in the pathogenesis of SAE. The level of CD11b was significantly higher in the SAE group than that in the Sham group (*P* < 0.01, **Figures 4A–C**), which was determined by immunofluorescence and western blot. The SAE + SCFAs group showed a lower level of CD11b compared with the SAE group (*P* < 0.01, **Figures 4A–C**). Measured by western blot, the level of COX-2 was remarkably increased in the SAE group compared with the Sham group, while SCFAs was significantly decreased it (*P* < 0.05, **Figures 4B, D**). The levels of IL-1β and IL-6 in the SAE group were significantly higher than those in the Sham group, while those in the SAE + SCFAs group were significantly lower than those in the SAE group (**Figures 4E, F**). The levels of JNK and NF-κB p65 were measured by western blot. The ratio of p-JNK/JNK and p-p65/p65 were remarkably increased in the SAE group compared with the Sham group, while SCFAs significantly decreased them (**Figures 5A–C**).

## Discussion

SCFAs are known to be actively involved in multiple brain disorders, while their roles in SAE remain unclear. In this study, SCFAs were shown to improve abnormal behavior, neuronal degeneration, and BBB impairment in the SAE mice, decrease excessive activation of microglia and production of pro-inflammatory cytokines, such as IL-1β and IL-6, increase the expression levels of tight junction-associated proteins, such as Occludin and ZO-1, and decrease the phosphorylation levels of JNK and NF-κB p65 in the brain of SAE mice, which elucidated its underlying molecular mechanism.

Behavioral impairment is one of the main features of SAE (21). A prospective case control study found that the behavioral results of children with SAE were significantly worse and there existed additional problems such as depression, conduct problems, psychotic behavior, and anxiety (22). Similarly, SAE mice showed impaired motor performance and decreased exploratory activity in the early stage (23) and a mouse model of sepsis induced by lipopolysaccharide displayed long-term depression and anxiety-like behavior (24). The improvement of cognitive dysfunction is a desirable target for therapies against SAE. Several SAE animals’ models have been established, such as CLP-induction and LPS-induction (24, 25). In this study, we established an SAE mice model induced by CLP, which is characterized by morphological and functional changes of the hippocampus and results in cognitive deficits. SCFAs could affect inflammation, emotional state, and cognition through the gut-brain axis (26, 27). Butyrate therapy could significantly improve learning and memory function by enhancing the expression of learning-related genes in an Alzheimer’s disease model mice (28). A recent study has shown that SCFAs’ regulation could reduce neuroinflammation and oxidative stress and improve cognitive ability in obese rats to some extent (29). Butyrate is used as an experimental drug in the model of neurological disorders such as depression, neurodegenerative diseases, and cognitive impairment (30). Acetate is a kind of SCFA with neuroprotective activity and could significantly improve the cognitive impairment of APP/PS1 mice (31). A study demonstrated that supplementation of SCFAs can lower the risk of neurodegenerative diseases (32). In this study, the SHIRPA protocol was used to assess behavioral impairment of SAE mice, which covers a variety of reflexes and basic sensorimotor functions (33, 34). Therefore, SCFAs could improve the behavioral impairment of SAE in mice.

The pathological changes and cognitive deficits occur simultaneously in SAE; brain pathological damage is also an important characteristic (35), which is manifested by impaired perception, consciousness, and cognition, resulting from multifactorial events (36). There is an association between brain damage and long-term psychological or cognitive disorders in SAE (37–39). According to reports, brain structures such as the neuroendocrine system, hippocampus, limbic system, frontal cortex, and brainstem of patients with sepsis are damaged (40); this brain damage might be related to psychological disorders including anxiety and depression, as well as memory and executive dysfunction, which is related to cognitive impairment (41). SCFAs have a certain effect on the changes of brain pathology (42, 43). In addition, Jaworska with his colleague found that butyrate could restore the number of neuronal cells by using a neonatal rat model of hypoxia-ischemia (44). Our previous study showed that SCFA butyrate could attenuate the pathologic changes and neuronal loss in the brain of traumatic brain injury model mice accessed by FJC staining (45). FJC staining is widely used for the specific detection of all degenerating mature neurons, including apoptotic, necrotic, and autophagic cells (46). In this study, SCFAs could decrease the numbers of FJC-positive neurons in SAE mice, suggesting that SCFAs could improve the pathological brain damage caused by SAE.

Notably, a key determinant of the development of SAE is the damage of the BBB (47), which protects the CNS from pathogens and toxicity (48). BBB dysfunction in sepsis and its association with clinical features (such as systemic inflammation) has been reported on (49). Clinical research has revealed cytotoxic or vasogenic edema as the most consistently reported MRI change in septic encephalopathy (50, 51). As is known, the BBB is a structural and biochemical barrier that regulates the entry of molecules from the plasma into the brain and preserves ionic homeostasis within the brain. The BBB is composed of microvascular endothelial cells which are closely linked together by TJs, including Occludin, claudins, and ZO-1 (52, 53). The functionally important part of the barrier is formed by TJs structures between the endothelial cells. Occludin and ZO-1 are key TJs in cerebral endothelial cells, which play an important role in modulating BBB functions (54, 55). BBB impairment showed decreased expression of tight junction proteins, such as occludin, ZO-1, ZO-2, claudin-3, and claudin-5 (56). Studies have shown that reducing the loss of ZO-1 and occludin proteins could restore the permeability of the BBB (57, 58). Furthermore, in an *in vitro* human cerebral endothelial cell model, pro-inflammatory cytokines and endotoxin resulted in a significant decrease in the expression of occludin (59). A recent study emphasized that SCFA butyrate played a biphasic role after stroke, reducing BBB permeability and oxidative stress in the brain (60). Our previous study showed that SCFA butyrate exerted neuroprotective effects by restoring the BBB in traumatic brain injury mice (61). In this study, the expression levels of occludin and ZO-1 were decreased in SAE mice and reversed by SCFAs, suggesting that SCFAs could restore BBB impairment caused by SAE.

SAE involves a number of mechanisms, in which neuroinflammation is critically involved in the pathogenesis of SAE (62, 63). Neuroinflammation is responsible for the dysfunction and massive apoptosis of brain cells, including microglial cells, neurons, and endothelial cells. Both peripheral inflammation and local inflammation are induced by activation of resident brain immune cells, such as microglial cells and astrocytes, and reportedly accounts for the induction of neuroinflammatory response and worse outcomes due to septic complications. The overactivation of microglia is involved in the progression of brain dysfunction by deteriorating the BBB. Microglia, a type of immune cell in the brain, could become activated upon pathological stimulation and be the basis of neuroinflammation (64). Microglia rapidly get activated in response to septic challenge and these cells produce substantial amounts of pro-inflammation factors, such as TNF-*α*, IL-6, and IL-1β, which could induce the amplified cerebral inflammatory response and thus exacerbate the brain injury (23). Overactivation of microglia is one of the main mechanisms of SAE (65). Immunohistochemistry showed that microglia were widely activated in an LPS-induced SAE mouse model (66), while inhibiting the overactivation of microglia could improve long-term cognitive behavior in CLP mice (67). Furthermore, the study showed that SCFAs alone or in combination could reduce the inflammatory response of microglia and regulate select microglial functions (68). Butyrate attenuated pro-inflammatory cytokine expression in microglia in aged mice (69), which could improve neuroinflammation. CD11b is a microglial marker secreted by activated microglia (70–72). During microglial activation, the expression of CD11b, the activated marker of microglia, is increased (73). A recent study revealed that SCFA butyrate has been shown to modulate the maturation of microglia (74). In this study, SCFAs could decrease the activation of microglia in SAE mice. It was reported that the levels of inflammatory cytokines, such as TNF-α, IL-1β, and IL-6, in the hippocampus of SAE mice were higher than normal (75, 76), and down-regulation of neuronal signals induced by neuroinflammation might be one of the causes of cognitive impairment in mice with sepsis-related encephalopathy (77). TNF-α, IL-1β, and IL-6 are proinflammatory cytokines, which could regulate a variety of physiological functions and play an important role in CNS (78). IL-1β could activate glial cells to trigger neuroinflammation and neurodegeneration (79) and might be involved in inducing neuronal apoptosis in cognitive dysfunction induced by neuroinflammation (80, 81). Moreover, it was revealed that TNF-α and IL-6 levels might be negatively correlated with cognitive function (82) and blocking of the IL-6 signaling pathway reduced cognitive flexibility (83). SCFAs could inhibit fructose-induced hippocampal neuronal inflammation and neuronal loss in mice (32) and inhibit a neuroinflammatory response (84). Liu et al. revealed that SCFAs decreased the production of IL-1 β and IL-6 in LPS-induced RAW264.7 macrophages (85). BBB disruption leads to the activation of microglial cells and the secretion of proinflammatory cytokines, which further aggravates brain permeability (86), while SCFAs could act on their own or in combination to reduce the inflammatory response of microglia (68), which would then in turn improve the BBB damage. In this study, our results showed that SCFAs could reduce levels of IL-1β and IL-6 and suppress the activation of microglia in SAE mice, suggesting that SCFAs could suppress the neuroinflammation of SAE.

NF-κB and JNK pathways mediate the transcription of various proinflammatory genes and play key roles in the neural inflammatory response (87, 88). In gram-negative sepsis, LPS induced activation of NF-κB, which translocated to the nucleus where it promotes transcription of inflammatory mediators, including COX-2 (89). In the brain tissues of the CLP-induced mice, the expression of NF-κB was enhanced (90). The study showed that exogenous SCFAs, especially butyrate, can block the activation of NF-B in diabetic glomerulonephritis mice (91) and Usami, M. et al. revealed that butyrate and propionate decreased the production of TNF-α in LPS-induced monocytes by inhibiting NF-κB activation (92). It was also indicted that a certain concentration of sodium acetate attenuates intestinal inflammation mainly by inhibiting MAPK activation and NF-κB phosphorylation (93). And Kobayashi et al. revealed that SCFAs, especially propionate, inhibited the phosphorylation of p38 and JNK in human renal cortex epithelial cells (94). In this study, SCFAs could significantly inhibit the phosphorylation of JNK and NF-κB p65, suggesting the effects of SCFAs against the neuroinflammation of AD *via* suppressing JNK and NF-κB signaling. In this study, our focus was on improving CLP-induced brain dysfunction. The molecular mechanisms underlying SCFA on SAE are still unclear. Currently, it is impossible to provide the overall mechanisms of SCFAs’ neuroprotective effects. SCFAs could cross the BBB and modulate CNS functions (15). SCFAs might interact with systemic immune cells, influence systemic inflammation, and then affect microglia involved in neuroinflammation. A further study to investigate the neuroprotective mechanisms of SCFAs on SAE is necessary.

In conclusion, this study elucidated that SCFAs could exert neuroprotective effects against SAE in mice. It is the first study to reveal the effects of SCFAs on attenuating behavioral impairment, neuronal degeneration, neuronal inflammation, and BBB impairment. Collectively, SCFAs might be a novel dietary supplement against SAE.

## Data Availability Statement

The original contributions presented in the study are included in the article/**Supplementary Material**. Further inquiries can be directed to the corresponding authors.

## Ethics Statement

The animal study was reviewed and approved by Animal Experimentation Ethics Committee of Wenzhou Medical University.

## Author Contributions

Z-QL and JL conceived and designed the experiments. YJ, YY, YT, SD, ML, GZ, and GH performed the experiments and conducted the statistical analyses. All authors contributed to the article and approved the submitted version.

## Conflict of Interest

The authors declare that the research was conducted in the absence of any commercial or financial relationships that could be construed as a potential conflict of interest.

## Supplementary Material

Supplementary Figure 1 Differential SCFAs’ identification. Seven kinds of SCFA and total SCFAs were detected **(A–H)**, and butyrate, valeric acid, hexanoic acid, and total SCFAs were significantly lower in SAE mice than those in the Sham group (*P* < 0.05). Acetate, propionate, isobutyric acid, and isovaleric acid were lower in SAE mice than those in the Sham group, however, there were no significant differences between the SAE group and Sham group (*P* > 0.05). Error bars indicate mean ± SEM. **P* < 0.05 versus Sham group, ***P* < 0.01 versus Sham group.

Supplementary Figure 2 Effect of SCFAs on the survival rate in SAE mice. Values are expressed as survival percentage.
