# Supplementary figures and images for: The Neuroprotective Effect of Short Chain Fatty Acids Against Sepsis-Associated Encephalopathy in Mice
Source: Front Immunol. 2021 Jan 28;12:626894. doi: 10.3389/fimmu.2021.626894 (PMC7876449; doi:10.3389/fimmu.2021.626894)

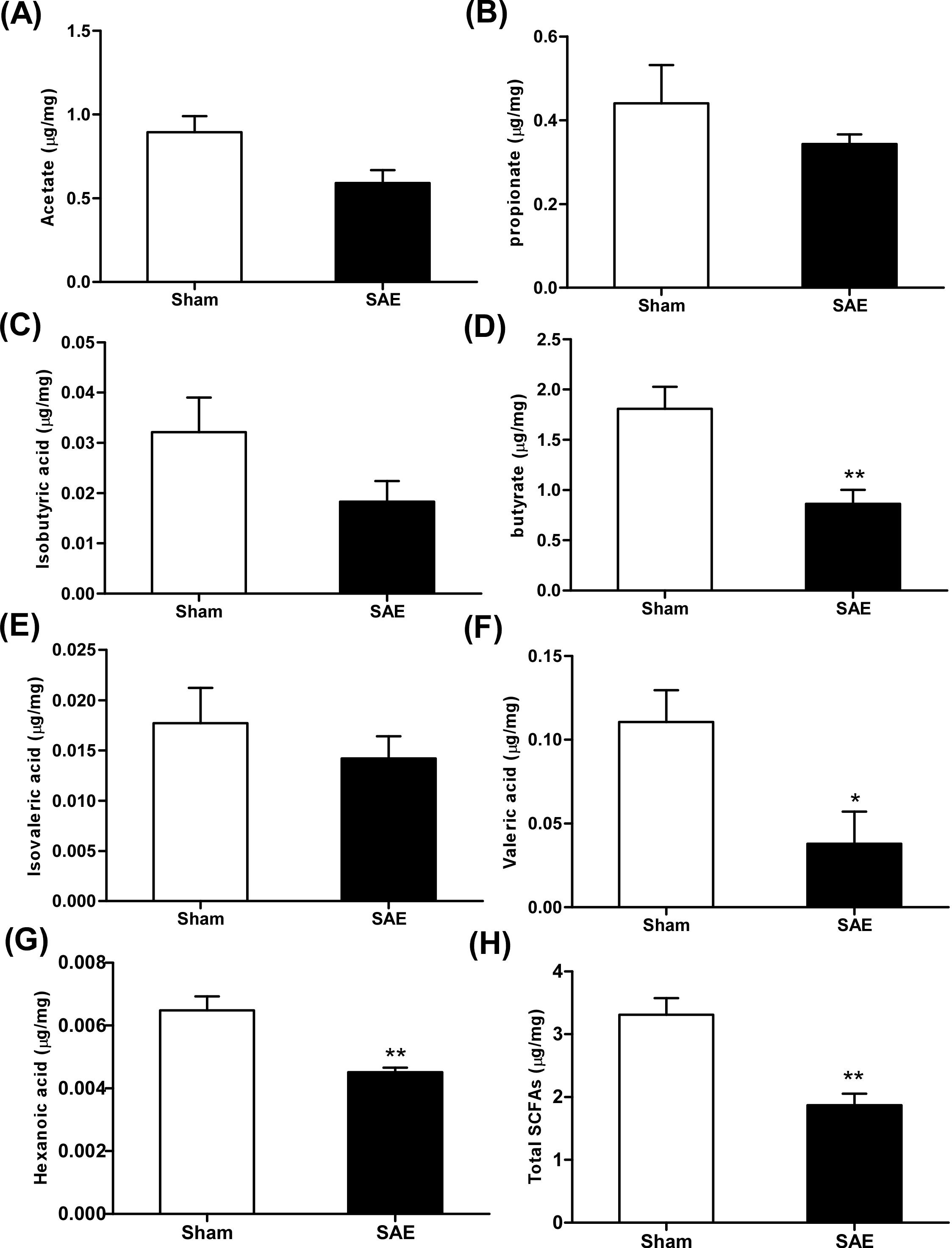

Supplement: Supplementary Figure 1 — Differential SCFAs’ identification. Seven kinds of SCFA and total SCFAs were detected (A–H), and butyrate, valeric acid, hexanoic acid, and total SCFAs were significantly lower in SAE mice than those in the Sham group (P < 0.05). Acetate, propionate, isobutyric acid, and isovaleric acid were lower in SAE mice than those in the Sham group, however, there were no significant differences between the SAE group and Sham group (P > 0.05). Error bars indicate mean ± SEM. *P < 0.05 versus Sham group, **P < 0.01 versus Sham group. [file Image_1.tif]

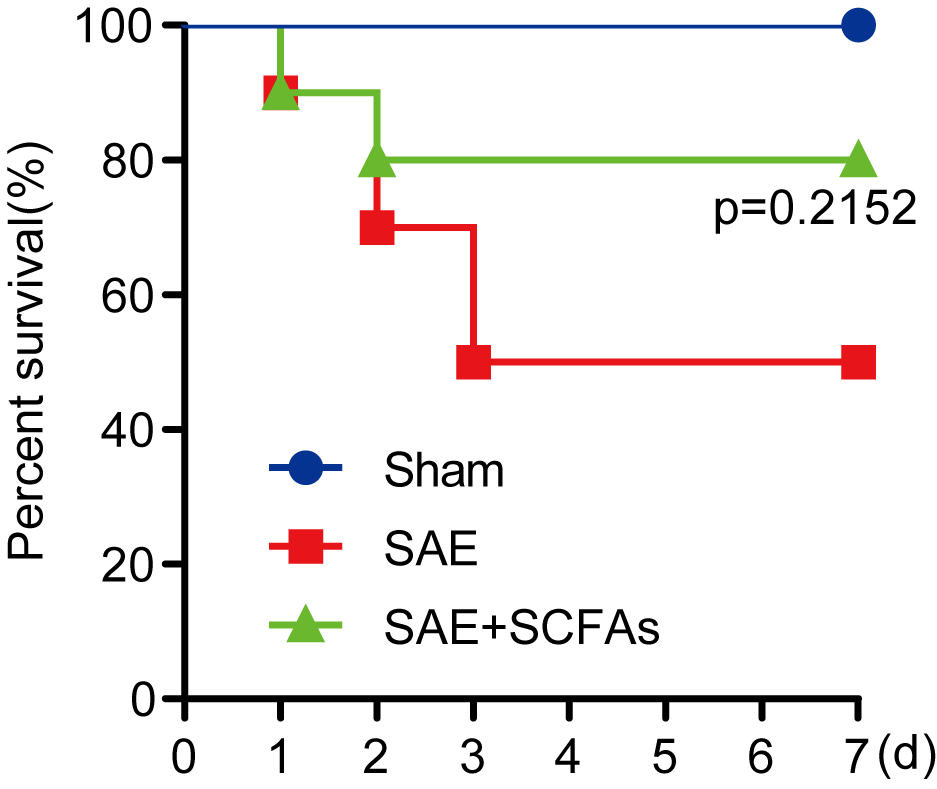

Supplement: Supplementary Figure 2 — Effect of SCFAs on the survival rate in SAE mice. Values are expressed as survival percentage. [file Image_2.tif]
